# Migrant Agricultural Workers’ Health, Safety and Access to Protections: A Descriptive Survey Identifying Structural Gaps and Vulnerabilities in the Interior of British Columbia, Canada

**DOI:** 10.3390/ijerph18073696

**Published:** 2021-04-01

**Authors:** Carlos Colindres, Amy Cohen, C. Susana Caxaj

**Affiliations:** 1 Emergency and Public Health Consultant, Vancouver, BC V7T 1A2, Canada; CarlosColindres@CHDEMconsulting.com; 2 Department of Anthropology, Okanagan College, Vernon, BC V1B 2N5, Canada; acohen@okanagan.bc.ca; 3 School of Nursing, Faculty of Health Sciences, Western University, London, ON N6A 3K7, Canada

**Keywords:** migrant agricultural workers, health and safety, social support, living conditions, barriers, service access

## Abstract

In this paper, we provide descriptive data that characterize the health, safety, and social care environment of migrant agricultural workers in British Columbia, Canada. Through the administration of surveys (*n* = 179), we gathered information in three domains: (1) living and working conditions; (2) barriers to rights, health, safety and advocacy/reporting; (3) accessibility of services. Our study confirms what predominantly qualitative studies and Ontario-based survey data indicate in terms of health, legal, and social barriers to care and protection for this population. Our findings also highlight the prevalence of communication barriers and the limited degree of confidence in government authorities and contact with support organizations this population faces. Notably, survey respondents expressed a strong intention to report concerns/issues to authorities while simultaneously reporting that they lacked the knowledge to initiate such complaints. These findings call into question government responses that task the agricultural industry with addressing access and service gaps that may be more effectively addressed by government agencies and service providers. In order to improve supports and protections for migrant agricultural workers, policies and practices should be implemented that: (1) empower workers to independently access health, social, and legal protections and limit workers’ dependence on their employers when help-seeking; (2) provide avenues for increased proactive inspections, anonymous reporting, alternative housing/employment and meaningful 2-way communication with regulators so that the burden of reporting is lessened for this workforce; (3) systematically address breaches in privacy, translation, and adequate workplace injury assessments in the healthcare system. Ultimately, the COVID-19 context has put into sharper focus the complex gaps in health, social and legal services and protections for migrant agricultural workers. The close chronology of our data collection with this event can help us understand the factors that have resulted in so much tragedy among this workforce.

## 1. Introduction

In 2019, over 50,000 temporary foreign workers came to Canada primarily under the Seasonal Agricultural Worker Program (SAWP) or the Agricultural Stream [1]. With over 72,000 work permits for migrant workers in agriculture issued, 46,707 were approved to SAWP workers, representing 65% of migrant agricultural workers in Canada [1]. The Agricultural Stream accounts for a smaller yet growing number of agricultural workers, recruited from various countries—such as Guatemala, the Philippines, and Thailand. In 2019, this group represented 23,796 migrant agricultural worker positions [1]. The SAWP brings in workers from Mexico, Jamaica, and eleven Caribbean countries [2]. The number of temporary agricultural workers coming to Canada has increased steadily since 2012, and in British Columbia (BC) 18% of farm labourers are migrant workers [1,3].

With the onset of the COVID-19 pandemic, the importance of migrant workers to Canadian agriculture has been increasingly recognized. Though for decades scholars have documented challenges these workers face accessing healthcare, social services, and legal protections, the pandemic has thrown into sharp focus the unique vulnerabilities that are faced by this workforce. Most tragically, in 2020, three migrant agricultural workers in Ontario lost their lives due to COVID-19, and another died prematurely due to a cardiovascular condition [4]. Thousands more have been infected while working on farms across Canada [5].

Prior research sheds light on key policy factors and conditions that make migrant agricultural workers more vulnerable to COVID-19. Key among these challenges are congregate and inadequate housing, employer mediation of healthcare, and dismissal and deportability of migrants with no opportunity for appeal [6,7,8,9]. Both as individuals and as members of the Migrant Worker Health Expert Working Group (MWHEWG), we have been active in pushing for policy changes that address the unique health needs of this population, both amidst and beyond the COVID-19 context. In this paper, we provide descriptive data that characterize the health and safety environment of migrant agricultural workers in British Columbia, Canada. Although initially intended to serve as baseline data for an intervention-based study, we report these results now with the aim of informing more timely policy and practice decisions that can better ensure the safety of this population during the ongoing pandemic and beyond.

## 2. Background: Migrant Agricultural Workers and Covid-19 

On 11 March 2020, the World Health Organization (WHO) declared a pandemic of the novel Coronavirus, SARS-COV-2 [10]. Based on large clusters of COVID-19 outbreaks around the world including in the United States of America, on 18 March the Federal government of Canada closed the Canada—U.S. border to all non-essential travel, banned the entry of foreign nationals and limited international passenger flights to four airports [11]. Two days later, the Canadian Federal Government granted an exemption for migrant agricultural worker travel in recognition of their critical role in the Canadian food supply chain [12].

The travel exemption took effect 26 March, and on 27 March Employment and Social Development Canada (ESDC), the agency responsible for the administration of the SAWP and Agriculture programs, released a set of guidelines outlining expectations for employers in light of COVID-19 [13]. The following week chartered flights carrying migrant workers from sending countries began arriving in Canada. On 14 April, the government announced $50 million to support employers in complying with the ESDC guidelines [14]. ESDC guidelines instructed employers to be responsible for the health of this workforce, including their 14-day self-isolation and housing standards (although provinces like BC assumed greater responsibility), access to medical care, and daily monitoring and documentation of symptoms. Given employers’ lack of knowledge of health and infection control, and ESDC’s limited acknowledgement of the unique vulnerabilities faced by this group, it was not surprising that these measures failed to protect migrant agricultural workers from contracting COVID-19 [15]. These vulnerabilities include a lack of personal protective equipment crowded living conditions, lack of paid sick time, and the temporary and conditional nature of migrant worker programs that discourage help-seeking because of significant pressure to be the ‘ideal worker’ [8,16]. Health barriers and challenges faced by migrant agricultural workers, although exacerbated by COVID-19, are long-standing and multi-faceted [8,17,18,19]. Yet very few quantitative studies exploring the prevalence of these issues have been undertaken.

### 2.1. Study Background and Objectives

The data we present in this report were gathered as a baseline for a longitudinal study of a pilot project aimed at increasing migrant agricultural workers’ access to health, social care, and legal protections. First initiated in the spring of 2019, this study aimed to address gaps in services for migrant agricultural workers and evaluate various indicators of effectiveness and sustainability. Staffed by an outreach worker and a legal advocate, this intervention helped farm workers navigate challenges they commonly face, including language and literacy barriers, lack of access to transportation, unfamiliarity with the Canadian health/legal system, and lack of knowledge of legal rights and entitlements. Given the greater risks of COVID-19 infection among this workforce in Canada [7], and the rapid development of policy concerning this population, we felt it necessary to present the data gathered thus far. Though these data will still serve their intended purpose in the future, a descriptive analysis is presented here to further inform health-care providers and policymakers as to various key indicators that both influence and illustrate the barriers migrant farm workers face to securing adequate health and safety. 

### 2.2. Review of the Literature 

#### 2.2.1. Living and Working Conditions

Migrant agricultural workers face several unique barriers to their health and wellbeing related to their living conditions and workplace. These barriers are largely consequences of living in employer-provided accommodations which are often crowded, substandard, and located on-farm far away from communities and support services. Workers face logistical barriers accessing basic services, such as internet, telephone services, and transportation. These barriers directly impact their health and wellbeing by limiting their ability to attain culturally-appropriate food, timely and independent medical care, and meaningful social support [6,20,21].

Migrant agricultural workers also face challenging work environments which include high levels of unsafe work, physiological distress, and workplace harassment [17,22]. Prior research has made evident that issues such as wage theft, workplace assault and discrimination, breaches of contract, and employer gatekeeping in healthcare, and medical repatriation occur among this population [23,24,25]. Yet aside from a few exceptions [19,26], attempts to quantify these incidents are rare, especially outside of Ontario. 

#### 2.2.2. Barriers to Rights, Health and Safety Advocacy, and Reporting

Prior research has also emphasized the limitations of a largely complaint-driven regulatory regime, in regard to employment standards, workplace health and safety protections, and housing [17,27,28]. The current process places the burden of reporting concerns on migrant agricultural workers rather than implementing a proactive monitoring and oversight regime. Prior studies indicate that these workers are often unaware of their legal rights in Canada. Additionally, the lack of legal services further complicates their ability to access justice, even when they are aware of their rights [29]. The risk to their employment, if they do report abuse, poses another significant barrier. It is well documented that workers who have reported health and safety concerns risk being fired or repatriated [17,26,30,31,32]. Finally, current methods for workers to report concerns are often inaccessible, intimidating, and lacking in 2-way communication necessary to instill confidence in reporting mechanisms. 

Similarly, this workforce is uniquely disadvantaged in pursuing their labour rights. In BC, an investigation regarding unfair labour practices found that returning Mexican workers who were union members had their visa reapplications unjustly denied not only by employers but also by government officials from Mexico who were responsible for assigning workers to employers [25]. All these factors have contributed to workers’ lack of faith in a system that is supposed to support them, and have further increased their fear of reporting health or safety concerns. The provision of support and services to this group may be further hindered by a conflict of interest that is created if a support organization serves both farm workers and their employers or employer associations. For instance, an organization that wishes to conduct independent outreach to address access barriers for migrant agricultural workers may be hesitant to do so out of concern that they will upset their farm operator clientele [8].

#### 2.2.3. Accessibility of Health and Social Services 

Within Canada, workers face many challenges when accessing healthcare and social services [6]. A lack of independent transport and isolation is a key barrier for accessing basic amenities and health services [25,33]. Given that workers often rely on employers or supervisors to access services and community supports, including medical care, receiving necessary follow-up medical care and maintaining communication with relevant support services is often difficult [19,34,35].

When they are able to access healthcare, migrant agricultural workers may face linguistic and cultural barriers. These barriers, combined with the fact that many have low levels of formal education, complicate their ability to advocate for their own rights or to access health services. Compounding this is the lack of independent and culturally relevant translation services [8,33]. Prior research documents that workers may be repatriated if they experience medical issues [26,31] and that in some cases clinicians may even participate in medical repatriation by developing a plan of care that is employer-focused rather than patient-oriented [8]. 

The transient and temporary nature of workers’ stay in Canada also creates barriers to workers asserting and following through on legal issues and complaints, including compensation of workplace injuries [35,36,37,38]. Furthermore, few supports are in place for this population, with many organizations limited in scope, funding, and capacity to meet their unique needs [39]. For example, many immigrant settlement agencies that may be well positioned to support this population are often only funded to support permanent residents and refugees [35] and healthcare workers may lack training to support this particular group [40]. So workers are limited both in terms of accessing necessary services and in receiving adequate follow-up to health or social challenges that are identified. 

## 3. Methods

### 3.1. Design

We carried out a cross-sectional baseline descriptive study based on self-reporting survey data.

### 3.2. Survey Development and Design

Key content for surveys was informed by the only prior quantitative health survey study conducted with migrant agricultural workers in Ontario [19], years of research conducted by C.S.C. and A.C. in the region that identified key challenges faced by this population [17,38,41], and public consultations with migrant agricultural workers (*n* = 235) and their support network (*n* = 40) conducted between 2017–2019 [8,35,42]. Surveys were translated/back-translated by a panel of experts in the field using simple syntax and included buffer questions, reverse coding, and clear instructions in an attempt to minimize sources of bias in the method. Strong attention was paid to tailoring the survey language for a population that often has low levels of literacy and formal education, and specifically for Central American respondents, taking into account that an Indigenous language, not Spanish, may be an individual’s first language [43]. Furthermore, our indicators of interest were simplified to take into account the limited knowledge this population typically has of the health, legal, and social service systems in Canada [35].

### 3.3. Data Collection

Data collection occurred from June to November 2019 in the interior of the province of British Columbia. Given the access and isolation issues faced by this population we employed a recruitment approach that enabled outreach through relationship-building and word of mouth. Thus, we used a snowball sampling technique, with initial respondents recruited in public spaces frequented by migrant agricultural workers. Recruitment for participants also occurred through referrals by front-facing staff. Surveys were administered or distributed by a research assistant during the initial meeting or at a mutually agreed upon time and place. In total, 201 potential respondents were approached, all agreed to be surveyed, and none declined after reading or being read the letter of information and consent form. For this analysis, 90.8% (*n* = 179) of the questionnaires were considered baseline surveys, and valid, with less than two missing items for each of the constructs.

### 3.4. Ethical Consideration

All surveys had a title page with instructions and consent form explaining the voluntary and confidential nature of the questions, and their sole use for research was verbally reviewed by a research team member before initiating the survey. In the case of recruitment through referral, individuals were first asked for consent to be contacted by the research team, after which the research team contacted the participant to explain the nature of the study and solicit consent to participate. Our team members made clear that no one would be denied help or services through our intervention, regardless of their willingness to participate in the study. The research project was reviewed and approved by research ethics’ boards at three institutions: University of British Columbia Okanagan (H18-02889) [Okanagan College] (19–005) and the University of Western Ontario (113627).

### 3.5. Survey Questions and Data

Survey questions assessing workers’ experiences were dichotomous yes no questions. Worker knowledge, attitudes, and perceptions were assessed with a 5-point Likert response scale of agreement ranging from ‘strongly agree’ (coded as ’1’) to ‘strongly disagree’ (coded as ’5’) with ‘agree’, ‘not sure’, and ‘disagree’ coded as ’2’, ’3’, and ’4’ respectively. The data are presented in this analysis trichotomized into ‘strongly agree/agree’, ‘not sure’, and ‘disagree/strongly disagree’ (see Appendix A).

## 4. Results

### 4.1. Demographics

Of the valid survey responses, 58 (32.4%) were female, 119 (66.5%) were male, with 2 responses missing (see Table 1). The age distribution of respondents was as follows: 22.9% were between the age of 25–35, 47.5% were between the age of 35–45, 16.8% were between the age 45–55, and 7.8% were >55 years of age. The most common country of origin for respondents was Mexico (86.6%), followed by Jamaica (10.6%). There was also one respondent from Micronesia, and four non-respondents on the question of country of origin. 

### 4.2. Living and Working Conditions

Survey responses provide a picture of migrant agricultural workers’ experiences and perceptions of safety and security in their workplaces and associated living environments. Participants reported a significant degree of workplace violence and harassment. To illustrate, 31.3% of survey respondents reported being discriminated against due to race, nationality in the past five seasons. Over 1 in 5 (21.8%) reported being threatened or intimidated by their employer, and 15.1% reported being assaulted by their boss or supervisor in the past five seasons. Many believed their job was tied to a fair amount of risk. Of the 179 participants, 114 (63.7%) stated that they believed their work in Canada put their health at some risk. Of these responses, 50 (27.9%) characterized the risk as large. Of all participants surveyed, 28 (15.6%) had been injured on the job over the last five seasons while working in Canada. Of those 28 who had been injured, 26 reported the injury to their supervisor, 16 went to the hospital for treatment, and 6 informed their consular officials of the injury. Injuries were reported to WorkSafeBC (the British Columbia Workers’ Compensation Board) in only three cases.

In addition, 90 (50.3%) respondents did not know who to contact to get help with translation, and 71 (39.7%) did not know who to contact to arrange transportation. Logically then, only 83 (46.4%) of respondents agreed, or strongly agreed, that they would be able to get the help they needed if a serious problem arose. Perhaps as a result of frequent experiences of violence, discrimination, and injury, compounded by their lack of access to independent transportation and support, workers reported little sense of belonging in their communities of residence while in Canada. A total of 102 respondents (57.0%) disagreed, or strongly disagreed, with the statement ‘I feel included in Canadian society while I work in Canada’.

### 4.3. Barriers to Rights, Health and Safety Advocacy/Reporting

Our data also shed light on temporary migrant workers’ knowledge, attitudes, and perceptions around health and safety concerns and advocating for their rights. Of the 179 migrant farm workers surveyed, 146 (81.6%) reported receiving training in workplace safety. Yet among those who had received training, the length of training varied greatly, with 40 (27.3%) reporting less than 20 minutes, 38 (26.0%) reporting 20 to 40 minutes, 48 (32.9%) reporting one hour, and only 20 (13.7%) reporting several hours of training. Roughly half (44.7%) of those surveyed (80) reported that they did not know what rights they had as workers in Canada (e.g., labour, housing, benefits, etc.).

Workers’ responses also revealed limited confidence in authorities and the regulatory system and in their capacity to access corresponding protections. In particular, 58 (32.4%) of the respondents disagreed, or strongly disagreed, that reporting their concerns to Canadian authorities would lead to greater protection for themselves or their coworkers, and 114 (63.7%) disagreed, or strongly disagreed, that their own government consular officials would take their concerns seriously. Perhaps as a result of this disenfranchisement, 136 (76.0%) of surveyed workers reported they did feel they had the same rights as Canadians while working in Canada. 

Despite the limited faith that respondents reported in these regulatory mechanisms, they reported high rates of intention to address problems that arose and seek support. For instance, 125 (69.8%) of workers said they would report workplace mistreatment or assault to Canadian authorities, and 146 (81.6%) said they would report unsafe or unhealthy work conditions to their consulate. However, 154 (87.0%) migrant farm workers disagreed with the statement ‘I know what steps I need to take to start a claim that I am entitled to make’.

Our analysis indicated a disjuncture between participants’ desire to seek justice or recover from illness or injury and their sense of a lack of infrastructure and support available to enable them to do so. To illustrate, 58 (32.4%) of workers disagreed, or strongly disagreed, that there were enough support people available to them to help them assert their rights. In total, only 27 (15.1%) respondents reported having received services from a support group during the entirety of their time in Canada. Support groups included both formal and informal organizations that provided services aimed specifically at migrant farmworkers such as government agencies, public service agencies (including healthcare organizations), and non-profit and civil society organizations. Despite these reports, 165 (97.2%) stated that they would continue to communicate or visit a support person until a serious problem was resolved if one was available. 

### 4.4. Accessibility of Health and Social Services 

Survey responses revealed some contextual factors at play in determining workers’ access, beliefs, and experiences with service delivery. Of those surveyed, 61 (34.1%) sought medical assistance due to illness or injury in the previous 5 years. Of those who sought medical assistance while in Canada, 12 had to pay out of pocket to access medical services and 44 had their boss or supervisor translate for them when receiving care. This high rate of employer participation in medical environments was influenced by the fact that 36 (81.1%) of the workers who accessed medical services depended on their boss or supervisor to translate for them, and were not offered another option in the form of an independent translator. 

A large percentage of migrant farmworkers (66 or 36.9%) did not know how to share information with medical professionals or support people. Many respondents had little faith in the health services that they would receive, with 47 (26.3%) stating they did not agree that they would receive the medical attention they needed in Canada, and 108 (60.3%) stating that they disagreed, or strongly disagreed, that they would receive the same quality of care as Canadians. Of particular significance, roughly half of survey participants (80 or 44.7%) disagreed, or strongly disagreed, that healthcare providers understood that their health issues could affect their employment, and 65 (36.3%) disagreed, or strongly disagreed, that their medical information would be kept confidential (i.e., that it would not be shared with others without their consent). Additionally, 68 individuals surveyed (38.0%) disagreed, or strongly disagreed, that the support service staff available, including medical staff, took the time to explain what the next steps were for their care or asserting their rights. 

## 5. Discussion

The COVID-19 context has underscored what researchers and community organizations have been stating for years: migrant agricultural workers are uniquely disadvantaged in accessing basic, appropriate, and comprehensive medical care [33,44,45]. Our study confirms many long-standing challenges documented in prior research, such as out-of-pocket expenses for medical services [8,38,46], and employer mediation in health care settings [8,19,47], particularly through a lack of an independent translator and default employer translation. Given the paucity of quantitative research in these areas, especially outside of Ontario, our findings, when taken together with research from across the country [16,19,33,37,44,48,49] confirm that these challenges are not specific to a particular region or province nor to a few individual cases.

### 5.1. Living and Working Conditions

Our findings are supported by previous studies that documented the prevalence of occupational injuries and illnesses among this population [17,19] and highlight the vulnerability of workers to workplace abuses. The prevalence of abuse casts doubt on the assumption that workers will be able to address concerns or make complaints to their employers or supervisors, as they are often the source of the abuse. Further complicating the issue is the lack of contact many workers have with community-based supports. One solution to addressing workers’ limited contact with support groups, and vulnerability to employer abuse could be greater adoption of independent and proactive oversight of housing and working standards for migrant agricultural workers. Certainly, relying on employers to ensure worker safety and security has proven to be ineffective and inappropriate [8,42]. 

### 5.2. Barriers to Rights, Health and Safety Advocacy and Reporting

Our data show the amount of time afforded to health and safety training is highly variable, suggesting a lack of standardization and potentially insufficient preparation to enter an incredibly hazardous sector. This, combined with limited contact with supports, and many workers’ limited knowledge of how to access independent translation and transportation, make it that much more difficult for them to seek help and access protections. In addition, workers reported limited knowledge of basic legal processes, including how to initiate a complaint or report and know their rights as workers in Canada. These issues combined may help to explain why migrant agricultural workers often do not report workplace violations or injuries [8,28,50], despite both the significant prevalence of workplace harms/abuses, and workers’ stated intention to report grievances. End of season evaluations that put in jeopardy a workers’ ability to return as well as fear of being deported can also impede workers from speaking out about their rights [17,32,51]. 

The high rate of workers’ intention to report concerns or issues to a formal authority stands in stark contrast to their limited confidence in these same authorities. Most did not express confidence that their consular officials would take their concerns seriously and a significant percentage did not believe that reporting to Canadian authorities would improve their access to protections. Prior research indicates that many workers have mistrust of their sending country government officials, in part because of the conflicting mandate of these officials’ roles (e.g., increasing participation in agricultural programs vs. protection of workers’ rights) [35,52]. Furthermore, these offices are very poorly staffed, with limited capacity and mandate to meet this population’s needs. Despite this limited resourcing, there is a general assumption that they are a key source of support for this population. For example, during the COVID-19 pandemic, the authors are aware of several health units that sought accompaniment and translation support from consular officials when visiting farms, rather than service providers, support organizations or professional translators.

Our data indicate that few migrant agricultural workers have confidence in Canadian authorities as a credible source of support when facing a complex issue. The Canadian enforcement regime has been criticized for years for failing to engage meaningfully with this group. Key concerns relate to a reliance on complaints rather than proactive unannounced inspections for enforcement, inaccessible communication channels that do not provide avenues for anonymous reporting or follow-up, and limited protocols and infrastructure to undergo comprehensive and culturally/linguistically appropriate engagement with workers [9,32,36]. These long-standing criticisms are substantiated by our research, since a large group of workers were not sure how to share information with support people when facing a problem. The fact that 86% of respondents reported not knowing how to file a formal complaint or report, and 76% did not feel that they had the same rights as Canadians underscores these challenges. 

The silver lining emerging from our data however, points to migrant agricultural workers’ high intention to communicate with support people to resolve an ongoing issue and to report workplace mistreatment or abuse to Canadian authorities. This suggests that with adequate reforms to the regulatory regime, workers will be able to meaningfully participate in asserting their rights and protections, given that they are already willing to do so. So while mistrust in authorities is justifiable when employer retaliation, including repatriation is possible [26,32], our data suggest that this mistrust could be understood as a gap in the current Canadian regulatory regime, rather than a reflection of an individual workers’ motivation to report. Reforms to the regulatory and inspection regime may be made more effective through increased funding and capacity for support actors who can help workers navigate the Canadian system [42] since less than half of workers agreed that they had enough help in this regard. Key to creating meaningful opportunities for workers to seek justice and compensation, is the need for the government to commit to funding alternative housing, employment, and accompaniment when a worker makes a claim [9].

### 5.3. Accessibility of Health and Social Services

Of the participants who sought medical care due to illness or injury, most were not given the option of independent interpretation, making their employer or supervisor their de facto translator. Lack of access to an independent translator is a concerning and a largely preventable challenge faced by migrant agricultural workers. Prior research indicates that medical paternalism and a prioritization of efficiency may be driving forces in clinicians not seeking suitable translators for migrant agricultural workers, even when there are available resources [8,53]. The COVID-19 pandemic has also emphasized that phone translation is not sufficient to ensure adequate healthcare follow-through for workers, especially when translators lack cultural and contextual knowledge of the group they are working with [9]. Yet the consequences of a lack of privacy in care as a result of employer mediation may include delays in medical treatment, complications in an individual’s medical trajectory, medical repatriation, and even employer retaliation [8,17,31,38,46]. Of great concern, ESDC’s initial response to COVID-19 further normalized and entrenched workers as conditional patients whose access to healthcare is contingent on employer discretion by downloading new health and safety responsibilities on to employers. 

Our research also highlights challenges workers faced communicating information to both health and social services. For instance, many participants expressed uncertainty about how to share information with medical and other support people and were concerned their medical information would be shared with others (e.g., employers, co-workers) without their consent. A large number of participants said they were not confident that their privacy would be ensured and proper consent obtained when seeking care, nor did they have confidence in follow-up communication from clinicians or other support people. This may speak to a larger issue which is that few front-line staff are well-versed on the particular context and experiences of this group [33,35,39]. A sense that clinicians lacked awareness of workers’ precarity was reported by many participants, with nearly 45% stating that they did not believe that healthcare providers understood that their health issues could affect their employment. This finding is especially troubling when taken together with the high percentage of workers who were injured and who did not report their injuries to WorkSafeBC, as it indicates that clinicians may be tacitly complicit in high rates of non-reporting [17].

Overall, participants did not express a lot of confidence in the Canadian healthcare system. As past research has shown, there are many gaps in healthcare delivery despite the model healthcare protections and benefits they are entitled to on paper [36]. Workers’ anticipation that the care they receive will be inferior to Canadians aligns with prior research documenting disadvantages workers face navigating the Canadian system. The fact that our research asked specifically about workers’ beliefs in this regard suggests that the Canadian healthcare system has failed to gain the trust of this population, and may have even instilled a sense of hopelessness or fear among workers who become sick or disabled in Canada. Prior scholarship with racialized migrant workforces suggests that a historical legacy of racism and current policies are contributing to various social vulnerabilities that ultimately affect this groups’ health status [54]. From this standpoint, migrant agricultural workers’ membership in society is delineated ideologically, and thus, gaps in policies that would ensure this population’s health and safety may be normalized and even more difficult to challenge [55].

## 6. Conclusions

We have described health and social challenges faced by migrant agricultural workers across three key domains: living and working conditions; barriers to rights, health and safety advocacy and reporting; accessibility of health and social services. Many of these challenges have been identified before, yet few studies have quantitatively documented their prevalence, especially outside of Ontario. Our research is also unique in quantifying health and social care barriers and workers’ limited confidence in government authorities. Notably, our study indicates that workers lack the knowledge and ability to report or initiate a claim, despite their stated intentions to do so. The close chronology of our findings with the emergence of COVID-19 on Canadian farms also suggests that key gaps in services and protections likely contributed to workers’ vulnerability throughout the pandemic. Upon the arrival of COVID-19 in Canada, advocates and experts raised the alarm, predicting that the long-standing challenges faced by this population would be exacerbated during this medical emergency [9]. Yet the federal government’s response failed to heed these warnings, and instead, helped perpetuate harmful assumptions and practices that have further endangered the health of migrant agricultural workers. These challenges, when understood within a historical context of marginalization of migrant workers, suggests that change must occur at both the discursive and practical level to prioritize the health and safety of this population.

## 7. Limitations and Considerations

The current study makes important and novel contributions to the understanding of various challenges faced by migrant agricultural workers, but its limitations must also be noted. Firstly, the study was based on a non-random sampling of individuals and those surveyed helped to recruit others interested in being surveyed. The snowball sampling method is vulnerable to sampling bias which may decrease the overall generalizability of the results. Secondly, self-reporting bias is possible given that data were obtained from a questionnaire. However, the congruency of the findings with literature in the field brings credibility to our findings and recommendations. Furthermore, the likelihood of reporting discrimination events due to social desirability bias and person-group discrepancy tends to under-reporting of those events [56,57]. Finally, at present, instruments that have established psychometric properties to measure migrant farmworkers’ experiences do not exist, limiting our data collection strategy. Our survey questions were developed taking into account the unique reporting barriers that may be encountered among this group and thus may be useful for others wishing to conduct research with this population. Nevertheless, the experiences we have documented indicate the need for further research, more current and valid measures used consistently over time, and multi-level analyses that connect policies to the experience of migrant agricultural workers and their health outcomes.

## Figures and Tables

**Table 1:** Demographic information.

Factors	*N*	%
Sex		
Male	119	66.5
Female	58	32.4
No response	2	1.1
Total	179	100
Age		
25–35	41	22.9
35–45	85	47.5
45–55	30	16.8
55–65	13	7.3
>65	1	0.5
No response	9	5.0
Total	179	100
Years worked in Canadian agriculture		
First season	21	11.7
2–3 years	30	16.8
4–5 years	36	20.1
6–10 years	27	15.1
11–15 years	46	25.7
16–20 years	18	10.0
More than 20 years	1	0.6
No response	0	0.0
Total	179	100
Country of Origin		
Jamaica	19	10.6
Mexico	155	86.6
No response	4	2.2
Micronesia	1	0.6
Total	179	100
Level of Education		
No school	1	0.6
Some primary school	14	7.8
Completed primary school	35	19.6
Some High School	114	63.7
Completed High School	9	5
Some college/university education	4	2.2
College/university degree or higher	1	0.6
No response	1	0.5
Total	179	100

## Data Availability

The data presented in this study are available on request from the corresponding author. The data are not publicly available due to privacy considerations.

## Appendix A

**Table A1:** Survey results of dichotomous questions concerning experience.

	Yes		No		Total	
	*N*	%	*N*	%	Count	%
Living and Working Conditions						
In the past 5 seasons, have you been discriminated against (because of your race, nationality, gender) by a boss or supervisor?	56	31.3	123	68.7	179	100.0
In the past 5 seasons, have you been threatened or intimidated by your boss/employer?	39	21.8	140	78.2	179	100.0
In the past 5 seasons, have you been assaulted by a boss or supervisor?	27	15.1	152	84.9	179	100.0
Barriers to Rights, Health and Safety Advocacy/Reporting						
Have you received any support services from these people?	21	11.7	158	88.3	179	100.0
Have you received training in workplace safety?	146	81.6	33	18.4	179	100.0
Accessibility of Health and Social Services						
In the past 5 seasons, have you seen a doctor or nurse for a medical problem (illness, injury, not feeling well)?	61	34.1	118	65.9	179	100.0

**Table A2:** Trichotomized survey results of Likert-scale questions concerning knowledge, attitudes and perceptions.

	Strongly Agree/Agree		Not Sure		Disagree/Strongly Disagree		Total Respondents	
	*N*	%	*N*	%	*N*	%	*N*	%
Living and Working Conditions								
I believe that my work in Canada puts my health at risk	114	63.7	5	2.8	59	33.0	178	99.4
I know who to contact to get help with transportation	107	59.8	1	0.6	71	39.7	179	100.0
I know who to contact to get help with translation	74	41.3	2	1.1	90	50.3	166	92.7
If I were to have a problem tomorrow, I would be able to get the help I need despite distance and transportation issues	83	46.4	3	1.7	93	52.0	179	100.0
I feel included in Canadian society while I work in Canada	77	43.0	0	0.0	102	57.0	179	100.0
Barriers to Rights, Health and Safety Advocacy/Reporting								
I know what rights I have as a worker in Canada (e.g., labour, housing, benefits)	92	51.4	7	3.9	80	44.7	179	100.0
I am confident that reporting concerns to Canadian authorities will help protect me and my co-workers	118	65.9	2	1.1	58	32.4	178	99.4
The representatives from my consulate will take my concerns seriously if I report something to them	59	33.0	6	3.4	114	63.7	179	100.0
I feel like I have the same rights as Canadians while working in Canada	41	22.9	1	0.6	136	76.0	178	99.4
If I experienced mistreatment in my place of work in Canada, I would tell Canadian authorities	125	69.8	3	1.7	51	28.5	179	100.0
If I experienced something at work that made me unsafe or unhealthy in Canada, I would report it to my consulate	146	81.6	3	1.7	30	16.8	179	100.0
I know what steps I need to take to start a claim that I am entitled to make (e.g., unemployment insurance, workplace injuries)	21	11.7	2	1.1	154	86.0	177	98.9
There are enough support people available to help me defend my rights	113	63.1	7	3.9	58	32.4	178	99.4
If I were to have a serious problem (e.g., housing, health care), I would continue to communicate or visit with a support person until the problem was resolved if one was available	165	92.2	9	5.0	5	2.8	179	100.0
Accessibility of Health and Social Services								
If I have a problem or need, I know how to share that information with support people(e.g., doctor, government staff)	111	62.0	2	1.1	66	36.9	179	100.0
While I work in Canada, I believe that I will get the attention/care that I need when I need it	124	69.3	7	3.9	47	26.3	178	99.4
I believe that I will get the same quality of care as Canadians when in hospitals or clinics	61	34.1	8	4.5	108	60.3	177	98.9
I feel that care providers understand that my health problems can affect my employment in Canada	90	50.3	9	5.0	80	44.7	179	100.0
Only I decide if my medical information is shared with others (e.g., boss, co-worker)	108	60.3	6	3.4	65	36.3	179	100.0
Support people take the time to explain what the next steps are for my care/problem	92	51.4	19	10.6	68	38.0	179	100.0
