# Correction: Colindres et al. Migrant Agricultural Workers’ Health, Safety and Access to Protections: A Descriptive Survey Identifying Structural Gaps and Vulnerabilities in the Interior of British Columbia, Canada. *Int. J. Environ. Res. Public Health* 2021, *18*, 3696

**DOI:** 10.3390/ijerph181910204

**Published:** 2021-09-28

**Authors:** Carlos Colindres, Amy Cohen, C. Susana Caxaj

**Affiliations:** 1 Emergency and Public Health Consultant, Vancouver, BC V7T 1A2, Canada; CarlosColindres@CHDEMconsulting.com; 2 Department of Anthropology, Okanagan College, Vernon, BC V1B 2N5, Canada; acohen@okanagan.bc.ca; 3 School of Nursing, Faculty of Health Sciences, Western University, London, ON N6A 3K7, Canada

In the original article [1], the last line of paragraph 2, page 7 is missing the word “not”, which changes the meaning of the sentence.

**This line should read:**

Perhaps as a result of this disenfranchisement, 136 (76.0%) of surveyed workers reported they did not feel they had the same rights as Canadians while working in Canada.

We would like to apologize for any inconvenience caused to the readers by these changes. 
